# Interplay of Antibody and Cytokine Production Reveals CXCL13 as a Potential Novel Biomarker of Lethal SARS-CoV-2 Infection

**DOI:** 10.1128/mSphere.01324-20

**Published:** 2021-01-20

**Authors:** Alexander M. Horspool, Theodore Kieffer, Brynnan P. Russ, Megan A. DeJong, M. Allison Wolf, Jacqueline M. Karakiozis, Brice J. Hickey, Paolo Fagone, Danyel H. Tacker, Justin R. Bevere, Ivan Martinez, Mariette Barbier, Peter L. Perrotta, F. Heath Damron

**Affiliations:** a Department of Microbiology, Immunology, and Cell Biology, West Virginia University, Morgantown, West Virginia, USA; b Vaccine Development Center at West Virginia University Health Sciences Center, Morgantown, West Virginia, USA; c Department of Pathology, Anatomy and Laboratory Medicine, West Virginia University School of Medicine, Morgantown, West Virginia, USA; d Department of Biochemistry, West Virginia University, Morgantown, West Virginia, USA; e West Virginia University Cancer Institute, Morgantown, West Virginia, USA; National Institute of Allergy and Infectious Diseases

**Keywords:** CXCL13, SARS-CoV-2, antibodies, cytokines

## Abstract

The SARS-CoV-2 pandemic is impacting the global population. This study was designed to assess the interplay of antibodies with the cytokine response in SARS-CoV-2 patients. We demonstrate that significant levels of anti-SARS-CoV-2 antibody to receptor binding domain (RBD), nucleocapsid, and spike S1 subunit of SARS-CoV-2 develop over the first 10 to 20 days of infection. The majority of patients produced antibodies against all three antigens (219/255 SARS-CoV-2^+^ patient specimens, 86%), suggesting a broad response to viral proteins. Antibody levels to SARS-CoV-2 antigens were different based on patient mortality, sex, blood type, and age. Analyses of these findings may help explain variation in immunity between these populations. To better understand the systemic immune response, we analyzed the levels of 20 cytokines by SARS-CoV-2 patients throughout infection. Cytokine analysis of SARS-CoV-2^+^ patients exhibited increases in proinflammatory markers (interleukin 6 [IL-6], IL-8, IL-18, and gamma interferon [IFN-γ]) and chemotactic markers (IP-10 and eotaxin) relative to healthy individuals. Patients who succumbed to infection produced decreased IL-2, IL-4, IL-12, RANTES, tumor necrosis factor alpha (TNF-α), GRO-α, and MIP-1α relative to patients who survived infection. We also observed that the chemokine CXCL13 was particularly elevated in patients who succumbed to infection. CXCL13 is involved in B cell activation, germinal center development, and antibody maturation, and we observed that CXCL13 levels in blood trended with anti-SARS-CoV-2 antibody levels. Furthermore, patients who succumbed to infection produced high CXCL13 and had a higher ratio of nucleocapsid to RBD antibodies. This study provides insights into SARS-CoV-2 immunity implicating the magnitude and specificity of response in relation to patient outcomes.

**IMPORTANCE** The SARS-CoV-2 pandemic is continuing to impact the global population, and knowledge of the immune response to COVID-19 is still developing. This study assesses the interplay of different parts of the immune system during COVID-19 disease. We demonstrate that COVID-19 patients produce antibodies to three proteins of the COVID-19 virus (SARS-CoV-2) and identify many other immunological proteins that are involved during infection. The data suggest that one of these proteins (CXCL13) may be a novel biomarker for severe COVID-19 that can be readily measured in blood. This information combined with our broad-scale analysis of immune activity during COVID-19 provides new information on the immunological response throughout the course of disease and identifies a novel potential marker for assessing disease severity.

## Abstract (precis)

The SARS-CoV-2 pandemic is continuing to impact the global population, and knowledge of the immune response to COVID-19 is still developing. This study assesses the interplay of different parts of the immune system during COVID-19 disease.

## INTRODUCTION

The SARS-CoV-2 pandemic has drastically affected life in the United States and across the globe. As of September 2020, more than 7 million people in the United States have been infected and over 200,000 patients have died (1). SARS-CoV-2 rapidly infected urban centers in California, New York, and other major cities across the United States that until recently were the main source of United States SARS-CoV-2 infections. Studies of anti-SARS-CoV-2 published in the first months of the pandemic were highly focused, with little exploration of the broader immune response in COVID-19 patients. Since then, several factors including elevated proinflammatory cytokines and others have been identified in SARS-CoV-2 pathology (2–7), but little is known about the interplay between cytokine production and the antibody response during SARS-CoV-2 infection. CXCL13 is a cytokine integral to germinal center formation (8–10) and has been used as a biomarker of an anti-infective antibody response (8). B cells are attracted to the germinal center via production of CXCL13 (11, 12) by follicular dendritic cells and T follicular helper cells (13, 14). Production of CXCL13 and B cell germinal center formation promote somatic hypermutation and affinity maturation of antibodies with virus-neutralizing function (8). CXCL13 production is quantifiable in human serum (15) and has not been characterized in the context of coronavirus infection in humans. In this respect, we sought to characterize the interplay of the antibody-mediated immunological response and the cytokine-mediated response to SARS-CoV-2 infection with a focus on CXCL13. We studied well-known markers of antiviral immunity including antibody production to the SARS-CoV-2 receptor binding domain (RBD), nucleocapsid (N), and spike s1 (S1) protein domains; Th1- and Th2-associated cytokine production; and CXCL13 production to characterize the immune profile of COVID-19 patients. Our study provides a broad view of the anti-SARS-CoV-2 immune response and reveals that CXCL13 may serve as a novel predictor of lethal infection in COVID-19 patients.

## RESULTS

### Inpatient anti-SARS-CoV-2 antibody levels.

**FIG 1:**
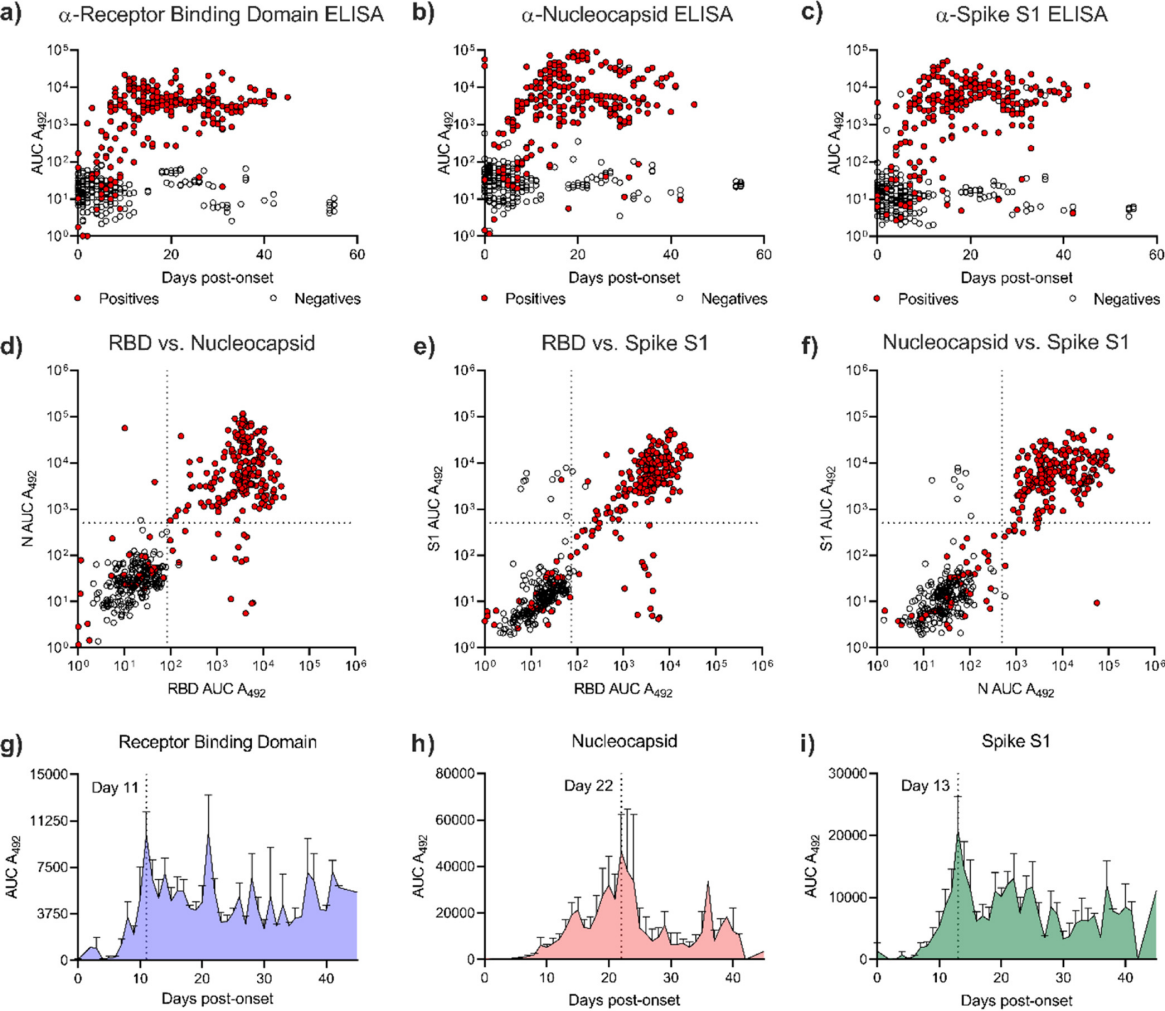
Anti-SARS-CoV-2 IgG response of SARS-CoV-2 inpatients. Antibody (IgG) levels of patient samples that tested PCR positive (red) or negative (clear) for SARS-CoV-2 to RBD (a), N (b), or S1 (c). Correlation of antibody levels to RBD versus N (d) or S1 (e). Correlation of antibody levels to N versus S1 (f). Antibody levels of anti-RBD (g), anti-N (h), or anti-S1 (i) produced by SARS-CoV-2-positive patients versus days after SARS-CoV-2 disease onset. *n* = 491 patient samples.

Antibody binding target and the timing of the antibody response are critical factors in mediating immunity. We evaluated levels of anti-SARS-CoV-2 antibody to 3 antigens (RBD, N, and S1) in 79 inpatients (see Table S1 in the supplemental material) by developing a novel rapid enzyme-linked immunosorbent assay (rapid-ELISA) technique. Our rapid-ELISA technology evaluates levels of IgG antibody against the SARS-CoV-2 RBD, N, and S1 proteins in approximately 1 h with greater than 99% accuracy (Table S2). Our survey of SARS-CoV-2-positive patients demonstrated that antibody (IgG) levels against RBD, N, and S1 proteins developed over the first 10 to 20 days post-symptom onset (Fig. 1a to c). When comparing levels of antibody to each antigen, we observed significant IgG levels against multiple antigens in the majority of patients tested (219/255 SARS-CoV-2-positive inpatient samples, 86%) (Fig. 1d to f, Fig. S1, and Data Set S1). To better understand the kinetics of the antibody response, we plotted IgG levels of every patient over time to RBD, N, or S1. Patients produced IgG against RBD rapidly after symptom onset with the peak IgG response occurring 10 days after symptom onset (Fig. 1g). Anti-S1 IgG levels escalated over a slightly longer period (13 days, Fig. 1i), and anti-N IgG production was slower than either anti-RBD or anti-S1 antibody production (22 days, Fig. 1h). Taken together, these data describe the breadth and timing of the IgG response to SARS-CoV-2 antigens.

10.1128/mSphere.01324-20.1 FIG S1 Rapid-ELISA results of all days post-symptom onset versus days 11+. Mean antibody responses of all patient samples (All) were compared to mean antibody responses occurring after 10 days postinfection (10+). Anti-SARS-CoV-2 IgG responses were not significantly different between all samples and samples 10 days after infection for all antigens. Both sample groups were significantly different from negative SARS-CoV-2 patients (−). Statistical analysis was assessed by one-way ANOVA followed by Sidak’s multiple-comparison test. ****, *P* < 0.0001 compared to negatives (−). n.s., not significant between All and 10+ groups. Download FIG S1, PDF file, 0.08 MB. Copyright © 2021 Horspool et al. 2021 Horspool et al. This content is distributed under the terms of the Creative Commons Attribution 4.0 International license.

10.1128/mSphere.01324-20.7 TABLE S1 Demographics of evaluated patient samples. Age, sex (proportion of female patients), patient outcome (proportion of surviving patients), and SARS-Cov-2 status of patients tested in this study. Download Table S1, PDF file, 0.03 MB. Copyright © 2021 Horspool et al. 2021 Horspool et al. This content is distributed under the terms of the Creative Commons Attribution 4.0 International license.

10.1128/mSphere.01324-20.8 TABLE S2 Accuracy of SARS-CoV-2 rapid-ELISA. Positive predictive value (PPV) or negative predictive value (NPV) was calculated for the rapid-ELISA technique of samples 10+ days postinfection. Positivity was assigned to samples that exhibited anti-N and anti-RBD antibody production. Download Table S2, PDF file, 0.06 MB. Copyright © 2021 Horspool et al. 2021 Horspool et al. This content is distributed under the terms of the Creative Commons Attribution 4.0 International license.

10.1128/mSphere.01324-20.9 DATA SET S1 SARS-CoV-2 supplemental data file: complete set of patient data recorded for analysis in this study. Download Data Set S1, XLSX file, 0.06 MB. Copyright © 2021 Horspool et al. 2021 Horspool et al. This content is distributed under the terms of the Creative Commons Attribution 4.0 International license.

### Antibody levels vary depending on patient population.

**FIG 2:**
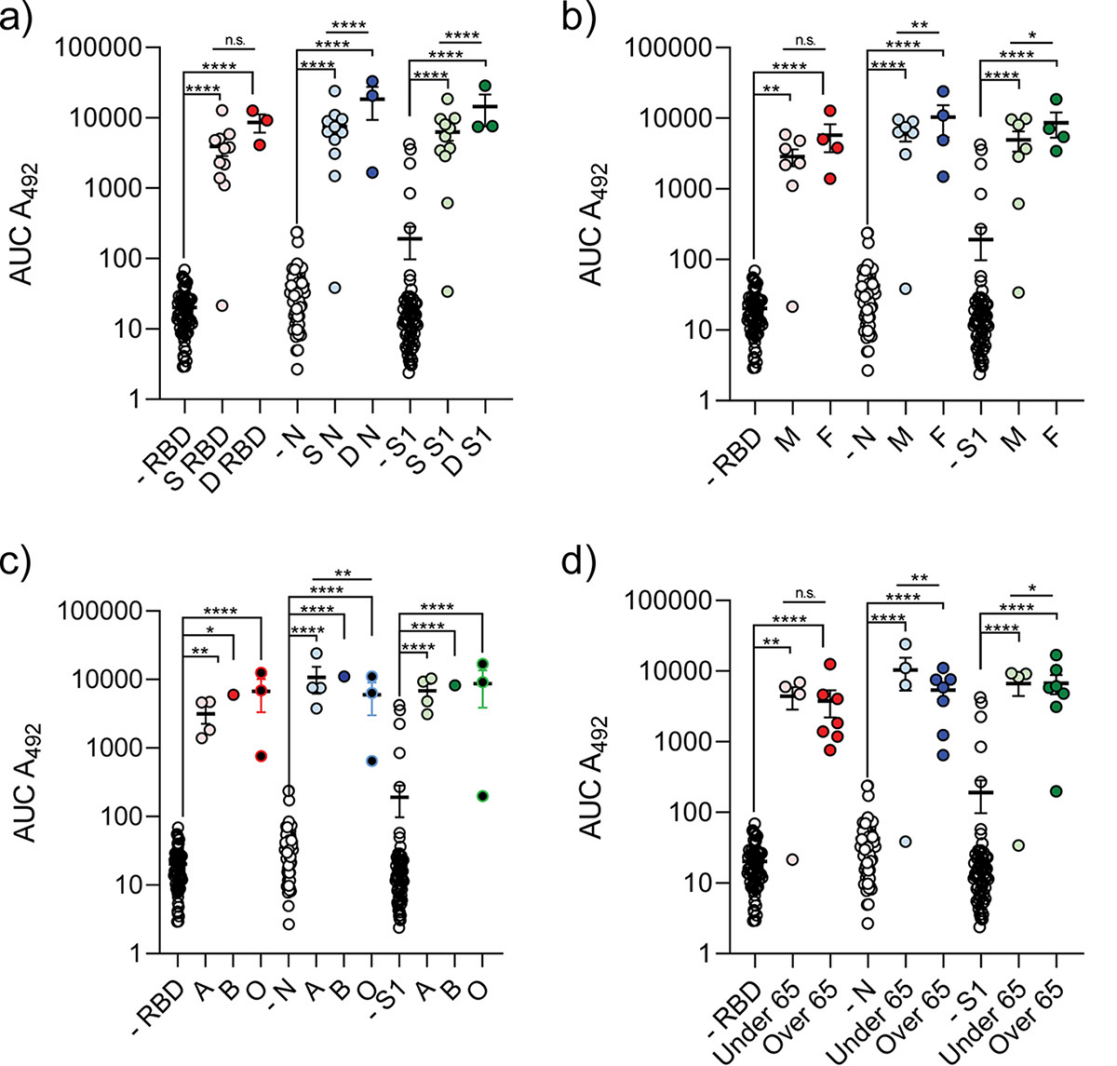
Patient lethality, sex, blood type, and age impact anti-SARS-CoV-2 antibody production. IgG levels of patients to RBD, N, and S1 separated based on patients’ lethality from SARS-CoV-2 infection (a). S, patients who survived SARS-CoV-2 infection; D, patients who did not survive infection; −, SARS-CoV-2-negative patients. Anti-SARS-CoV-2 antibody production of surviving patients separated based on sex (b), blood type (c), and age (d). Statistical analysis was completed by one-way ANOVA followed by Sidak’s multiple-comparison test. *, *P* < 0.05; **, *P* < 0.01; ***, *P* < 0.001; ****, *P* < 0.0001; n.s., not significant. *n* = 77 patients, *n* ≥ 3 patients per group.

Antibody responses are typically different depending on patient demographics and have implications for population-wide immunity. To understand the anti-SARS-CoV-2 IgG response in different populations, we analyzed patient groups based on sex, patient mortality, blood type, and age against anti-RBD, anti-N, or anti-S1 antibody levels. Patients who did not survive SARS-CoV-2 hospitalization produced significantly more antibodies to SARS-CoV-2 N and S1 than patients who survived infection (Fig. 2a). To accurately assess differences in antibody production independently of disease outcome, we quantified anti-SARS-CoV-2 IgG production in patients who survived infection grouped by biological sex, blood type, and age. We determined that, in our cohort, females produced significantly more antinucleocapsid and anti-S1 IgG than males (Fig. 2b). We also observed that blood type was significantly associated with anti-SARS-CoV-2 IgG production (Fig. 2c). Blood type A^+^ patients produced the lowest quantities of anti-RBD and anti-S1 IgG. Conversely, O^+^ patients produced reduced anti-N and elevated anti-RBD/S1 IgG relative to A^+^ or B^+^ patients. Previous studies have identified that age impacts antibody production to SARS-CoV-2 (16, 17). Our study demonstrates that antibody production against RBD is similar between individuals below and those above the age of 65 (Fig. 2d). In contrast, antibody production to N is increased in patients under the age of 65 and anti-S1 IgG levels are increased in patients over the age of 65. These trends are evident when examining Pearson correlations between age and anti-SARS-CoV-2 IgG production for each antigen which describe a weak association between age and anti-SARS-CoV-2 IgG production (Fig. S3). Overall, these data document a significant impact of patient demographics on anti-SARS-CoV-2 antibody production.

### Changes in SARS-CoV-2 patient cytokine responses correlate with disease severity.

**FIG 3:**
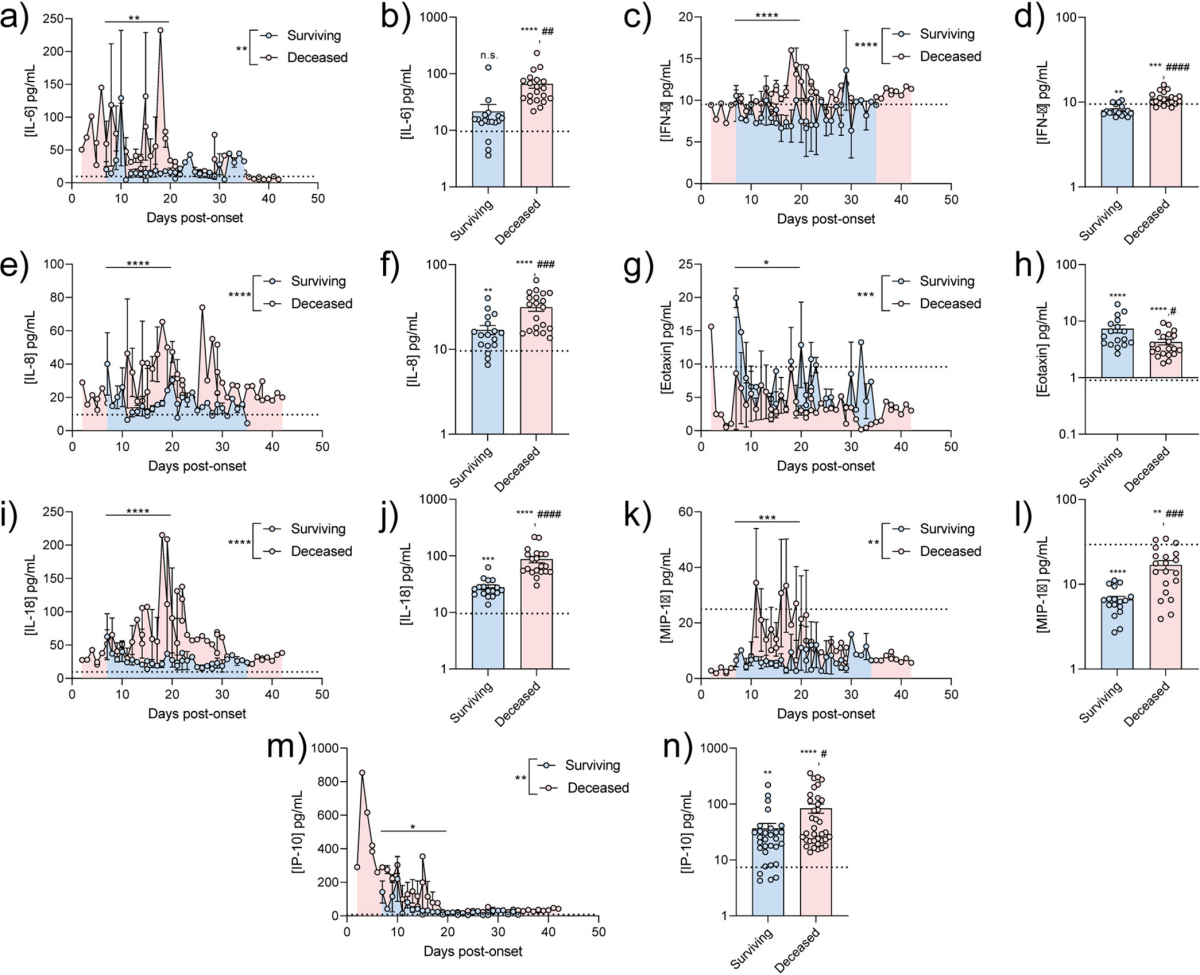
SARS-CoV-2 patient cytokine profile is impacted by disease severity. Patient cytokine profiles and concentrations for IL-6 (a and b), IFN-γ (c and d), IL-8 (e and f), eotaxin (g and h), IL-18 (i and j), MIP-1α (k and l), or IP-10 (m and n) were determined over time by Luminex technology. *n* ≥ 3 patient cytokine profiles were combined, and cytokine concentrations per day between days 7 and 21 were averaged. Technical replicates were averaged in GraphPad Prism prior to patient data combination. Full cytokine profiles for a surviving patient (i) or deceased patient (j). Surviving, SARS-CoV-2^+^ patients who survived infection; deceased, SARS-CoV-2^+^ patients who did not survive infection. Statistical significance was assessed between total average cytokine concentrations across days (reported in longitudinal graph legend) and between cytokine concentrations averaged between days 7 and 21 (reported on longitudinal figures and histograms). Significance between bars was determined with a two-tailed Welch *t* test. Significance against average cytokine concentration of healthy controls (dotted line) was assessed with a one-sample *t* test. *, *P* < 0.05; **, *P* < 0.01; ***, *P* < 0.001; ****, *P* < 0.0001; n.s., not significant. #’s represent significance between bars on histograms.

Antibody production represents the antigen-specific response to pathogens but is only one facet of immunity. We examined the broader immunological response to SARS-CoV-2 infection by quantifying the production of cytokines involved in a representative subset of SARS-CoV-2 or healthy patients. SARS-CoV-2 patients exhibited significant increased proinflammatory cytokine production (interleukin-6 [IL-6], IL-8, IL-18, gamma interferon [IFN-γ]) and altered chemotactic cytokine production (IP-10, MIP-1α, and eotaxin) relative to noninfected individuals (Fig. 3). Of the SARS-CoV-2-infected patients, mortality was associated with increased IL-6, IL-8, IL-18, and IP-10 production. Patients who succumbed to infection also demonstrated intermediate production of eotaxin relative to surviving SARS-CoV-2 patients and healthy individuals. We observed no statistically significant differences in several other measured cytokines between healthy and SARS-CoV-2-positive patients. However, we observed that lethal SARS-CoV-2 infection was associated with significantly decreased IL-1β, IL-2, IL-4, IL-12, RANTES, tumor necrosis factor alpha (TNF-α), GRO-α, and MIP-1α or increased IFN-γ (Fig. S4). All patient cytokine profiles studied are documented in Fig. S6. Together, these data demonstrate that SARS-CoV-2 patients exhibit an increased proinflammatory and chemotactic response with distinct profiles associated with patient mortality.

### CXCL13 as a novel predictive tool of lethal SARS-CoV-2 infection.

**FIG 4:**
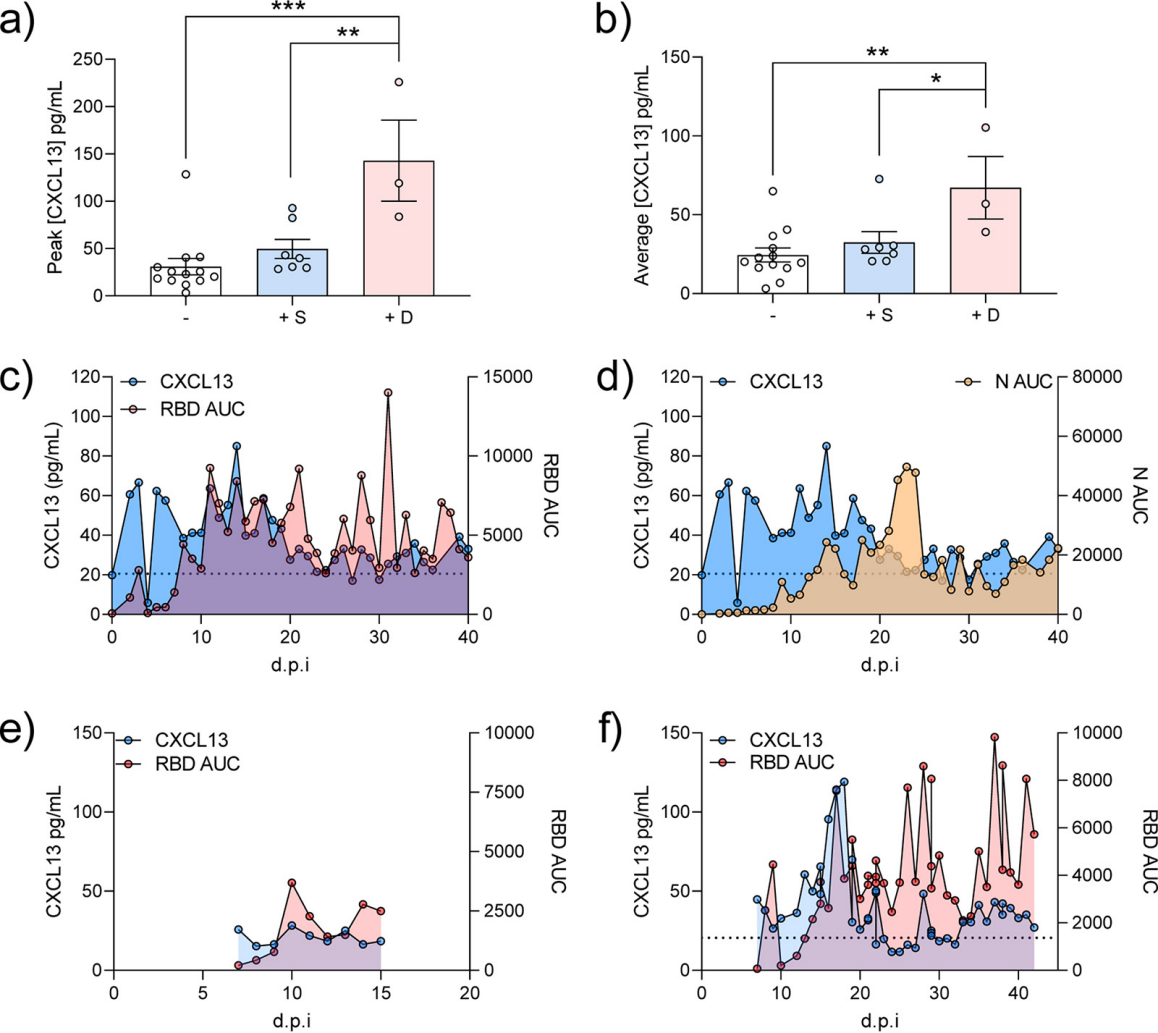
CXCL13 as a novel biomarker for lethal SARS-CoV-2 infection. CXCL13 peak (a) or average (b) concentration was measured in SARS-CoV-2-positive and -negative patients. CXCL13 production by SARS-CoV-2 production is compared to anti-RBD (c) or anti-N (d) IgG quantity over the course of patient disease. Examples of a surviving patient producing low CXCL13 and low anti-RBD IgG response (e) or deceased patient producing high CXCL13 and high anti-RBD IgG response (f). Statistical significance was assessed with an ordinary one-way ANOVA followed by Tukey’s multiple-comparison test. *, *P* < 0.05; **, *P* < 0.01; ***, *P* < 0.001; ****, *P* < 0.0001; n.s., not significant. *n* ≥ 3 patients per group.

Infectious disease stimulates germinal center formation promoting high-affinity antibody production (8, 10, 18–20). This response is critical for eradicating many pathogens. As many SARS-CoV-2 patients produced robust antibody responses to multiple antigens, we hypothesized that germinal center formation would be increased in these patients. To quantify this, we measured the serum concentration of CXCL13, a critical mediator of germinal center formation and a biomarker of this immunological response (8, 10, 18, 19). We observed that there was a significant increase in peak and average production of CXCL13 in positive patients relative to negative SARS-CoV-2 patients (Fig. 4a and b). CXCL13 production primarily correlated with peak antibody production to RBD and S1 antigens across SARS-CoV-2-infected patients (Fig. 4c and d). Additionally, we observed that CXCL13 production was significantly increased in patients who did not survive SARS-CoV-2 infection compared to those who did (Fig. 4a and b). When we compared antibody and CXCL13 production levels based on patient survival over time, we observed that patients who did not survive SARS-CoV-2 infection (representative patient profile in Fig. 4f) exhibited a sustained increase in antibody and CXCL13 production relative to surviving patients (representative patient profile in Fig. 4e). A full comparison of CXCL13 to anti-SARS-CoV-2 IgGs is provided in Fig. S5. These results suggest that CXCL13 and intense germinal-center-driven antibody responses are likely associated with lethal SARS-CoV-2 infection.

## DISCUSSION

Understanding the breadth of the immune response to SARS-CoV-2 infection may be critical to better manage SARS-CoV-2 and prevent it from permeating vulnerable communities on a local and global scale. Initially, we used our rapid-ELISA to rapidly assess anti-RBD, anti-N, and anti-S1 antibody production in PCR-positive or PCR-negative SARS-CoV-2 inpatients admitted to a West Virginia (WV) hospital. Antibody production against multiple SARS-CoV-2 antigens developed over the course of 20 days postinfection in a manner similar to other studies (21–25). Interestingly, IgG antibody production to N increased over a longer period than antibodies against RBD, or the S1 domain. This could be due to a variety of factors including antigen immunodominance (26, 27), incongruent antigen processing and availability (28, 29), differences in antibody utility and turnover, or prior exposure to similar RBD/S1 antigens of other coronaviruses. Theoretically, as N is not expressed on the viral surface, B cells producing antibodies against this antigen may not be selected for as rapidly as those that are specific to the RBD or S1 antigens and may not possess neutralizing function. As infection worsens, more cells lyse. This may increase the local concentration of free nucleocapsid available for antigen processing and presentation, particularly in lymphoid tissue (30). In this respect, a more robust antibody response to nucleocapsid later in infection may be due to increased cellular damage. This may initiate a positive feedback loop where infected cells lyse and release nucleocapsid, which induces a less functional antinucleocapsid antibody response that fails to alleviate the cell lysis. More evidence is required to support these hypotheses, but these are interesting paradigms to consider in the context of anti-SARS-CoV-2 immunity.

Lethal SARS-CoV-2 infection is significantly correlated with higher antibody production (16, 22, 25) and is described further in this study. We observed significant differences in IgG levels in deceased patients infected with SARS-CoV-2 in a limited number of patients. Larger-scale studies will be critical in evaluating these trends in more detail. In analyzing antibody production between patient demographics further, it was important to eliminate increased antibody production due to lethal infection as a source of bias. As such, our analyses presented here describe IgG production of SARS-CoV-2 survivors grouped by demographic. There are a multitude of studies reporting differences in IgG production between demographics, including trends in anti-SARS-CoV-2 antibody production between sexes (16, 17, 31–33), a correlation of genetically encoded blood type with SARS-CoV-2 immunity (34), and variability in antibody production in the aging population (16, 17). From these prior studies and others (35, 36), it is known that biological sex can impact antibody production during infection. We observed this phenomenon when quantifying sex-specific anti-N and anti-S1 IgG production. The antiviral response is mediated in part by Toll-like receptors which are differentially regulated between the sexes (37, 38). A higher frequency of anti-SARS-CoV-2 IgG in females would suggest an increased response to the virus, which may alter neutralizing capacity, an idea that has not been thoroughly evaluated to date. Our data exhibited a modest and nonsignificant difference in antibody production between sexes. As a result, we do not consider biological sex to be a major contributor to anti-SARS-CoV-2 antibody production.

It is documented that red blood cell phenotypes can influence microbial pathogenesis as antigens can function as receptors and/or coreceptors for pathogenic organisms (39). Historically, an association was identified between ABO type and pathogen infectivity during the SARS-CoV Hong Kong hospital outbreak in 2003; during that outbreak a small cohort of type O health care workers showed significantly decreased odds of infection relative to health care workers with other blood types (39, 40). An additional study demonstrated that antibodies against the A blood type antigen can inhibit SARS-CoV spike protein binding to ACE2 (41). Although the underlying mechanism relating blood type to SARS-CoV-2 pathogenesis remains unclear, it appears there may be a relationship between ABO blood type and coronavirus infection. Recent data identified the 9q34.2 locus (ABO blood group locus) as potentially involved in susceptibility to COVID-19 respiratory failure with evidence that type A phenotypes are at higher risk while type O phenotypes are partially protected (42). The data generated in these studies show an interesting pattern that may reinforce blood-type-related outcomes in severe disease due to a previously unreported association with the level and type of antibody response. As seen in Fig. 2c, the relative quantity of anti-RBD and anti-S1 antibodies was highest among type O and B individuals and lowest in type A individuals while the opposite is true of anti-N antibodies. This is further accentuated by evaluating the ratio of anti-RBD or anti-S1 to anti-N in our patient cohort, which shows that higher N/RBD or N/S1 ratios are associated with poor prognosis (see Fig. S5 in the supplemental material). These data suggest that there are connections between blood type, antibody levels, and prognosis, but broader analyses of the global population are required to draw meaningful conclusions from these data.

The aging process is associated with decreased T-cell functionality (43), resulting in hyperactive B-cell proliferation that does not confer immunity (44). We discovered a weak association between age and anti-SARS-CoV-2 IgG production in this study. Increased anti-S1 and decreased anti-N IgG levels may be a function of antigen availability. To speculate, if elderly patients have higher viral loads due to decreased remediation of virus, this would increase the relative abundance of surface-exposed antigens (RBD and S1), but not necessarily hidden antigens (N). Increased antibody production would therefore predominantly occur to RBD and S1, and not N. Other challenges are associated with studying this population, including copresentations of multiple diseases which complicate this analysis. Regardless, our study has identified several patient demographics associated with differences in the anti-SARS-CoV-2 antibody response.

The antiviral immune response depends on a variety of signaling pathways mediated by cytokines and chemokines. Many of the proinflammatory cytokines associated with the antiviral response are upregulated in patients with lethal SARS-CoV-2 infection in our study. IL-6, IL-8, IL-18, and IFN-γ are known proinflammatory cytokines that aid in the antiviral response and have been identified in other studies of SARS-CoV-2 patients (2, 45–49). These cytokines are considered part of the “cytokine storm” notorious for inducing localized tissue damage, which may explain the relative increase of these cytokines in deceased patients. In general, the production of these cytokines in SARS-CoV-2 patients was similar to that in individuals with other lethal viral infections (50–52).

Chemotaxis is another critical component of antiviral immunity, and several chemotactic mediators were increased in patients from our study. IP-10 is a chemotactic agent that was increased 10-fold in SARS-CoV-2 patients and even more so in deceased SARS-CoV-2 patients. IP-10 is protective in SARS infection (53, 54), suggesting that this may be a critical component of anti-SARS-CoV-2 immunity. In a broader sense, this chemotactic response likely functions by inducing chemotaxis of phagocytic immune cells and activated T cells similar to other viral infections (55). Several other chemoattractive mediators with similar function were upregulated in SARS-CoV-2 patients, revealing a systemic increase in leukocyte recruitment (Fig. 3 and Fig. S4). One functional outlier in this analysis was eotaxin, which is an interesting chemokine with broader functional capabilities. We discovered increased eotaxin production in SARS-CoV-2 patients. Eotaxin was increased or similar to that in healthy patients during SARS-CoV-2 infection in other studies (46, 56). Eotaxin is typically involved in eosinophil recruitment, which can result in pulmonary damage (57). This chemokine is upregulated during viral infection (58) and can inhibit certain viral infections, such as HIV (59). As patients who survived infection produced significantly more eotaxin than patients with lethal infection, it is possible that eotaxin provides a double-edged function in SARS-CoV-2 immunity.

**FIG 5:**
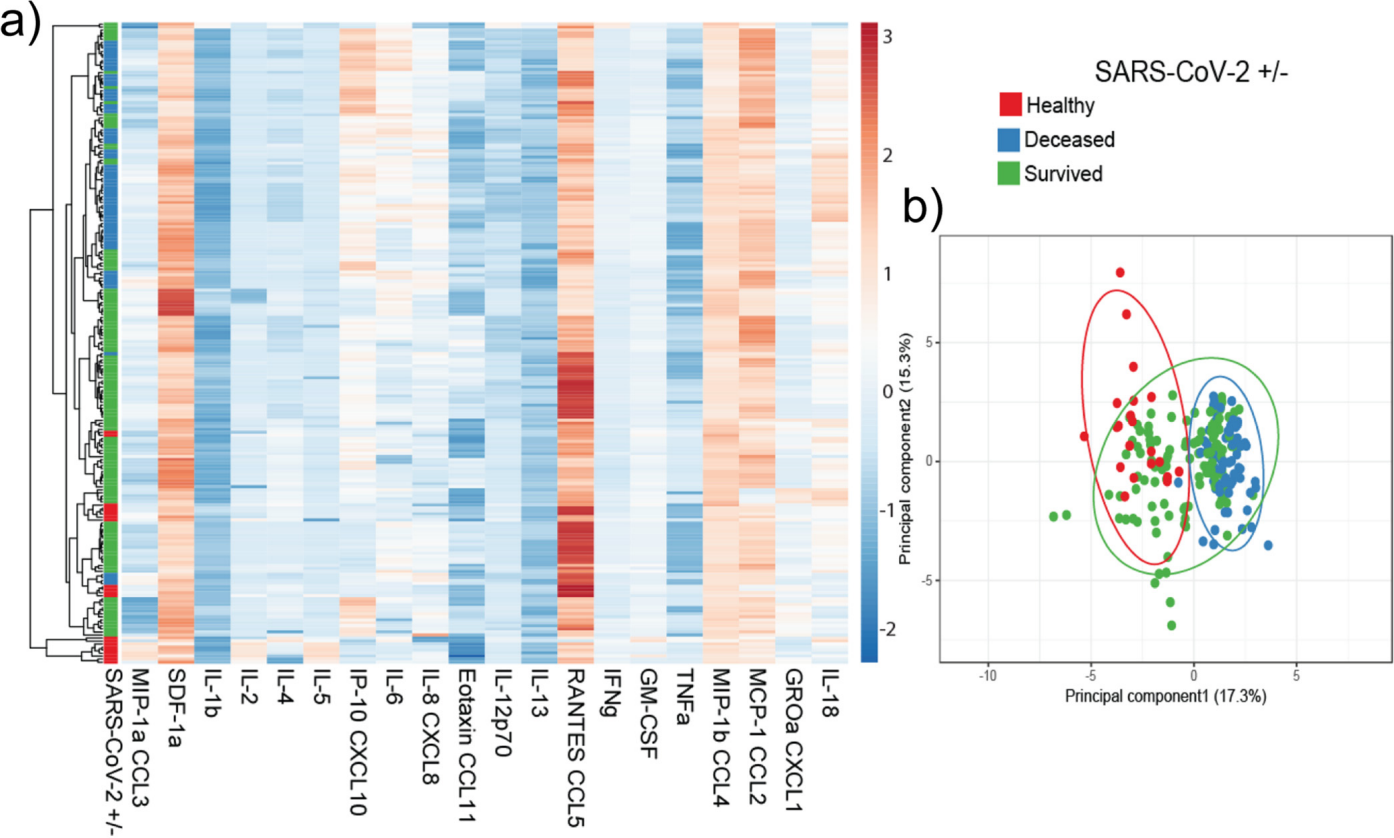
Principal-component analysis of anti-SARS-CoV-2 immunological responses. Heatmap (a) and principal-component analysis (b) of all patient samples including 20 cytokine concentrations clustered using ClustVis (63).

Surprisingly, we did not observe changes in production of a number of other cytokines that are involved in the general antiviral response (i.e., TNF-α [Fig. S4 and S6]). Although this was the case, the noticeable decreases in IL-1β, IL-2, IL-4, IL-12, IL-13, RANTES, TNF-α, and GRO-α observed in patients with lethal SARS-CoV-2 infection suggest that lethal infection results in an exhausted immune response (60, 61). When considering the overall cytokine response to SARS-CoV-2 in conjunction with the anti-SARS-CoV-2 antibody response, it is clear that there are distinct phenotypic clusters of healthy patients, surviving patients, and patients with lethal infection (Fig. 5). In this respect, these analyses paint a more definitive picture of the anti-SARS-CoV-2 cytokine response. Despite the significant results of this study, these data should be evaluated in broader context as patient demographics, treatment plan, and course of infection likely play a role in differences in cytokine production between patients and studies. Large-scale analyses of cytokine production on a population-wide scale would likely be necessary to fully understand the cytokine profile of anti-SARS-CoV-2 immunity.

**FIG 6:**
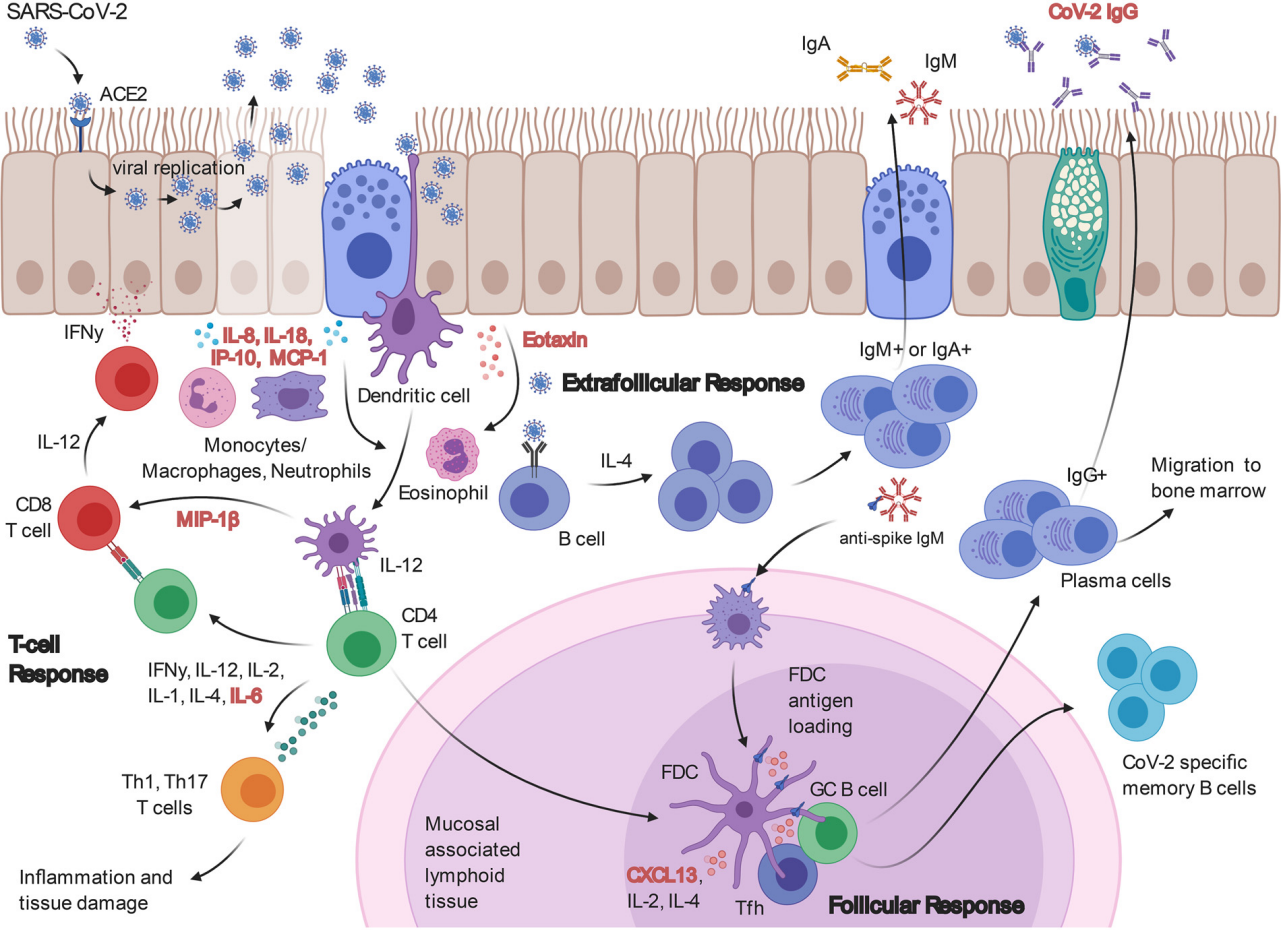
Overview of SARS-CoV-2 immunity. A schematic of the findings provided in this study (increased immunological markers highlighted in red) in the context of anti-SARS-CoV-2 immunity.

Antibody maturation signaling has not been investigated in the context of SARS-CoV-2. We assessed the activity of the antibody maturation pathway by measuring CXCL13 concentrations in the serum of SARS-CoV-2 patients. Increased CXCL13 in SARS-CoV-2 patients may indicate heightened germinal center activity (8) and affinity maturation of anti-SARS-CoV-2 antibodies. The significant increase of CXCL13 in patients with lethal disease suggests this may be an emergency response to uncontrolled infection. It is possible that sustained infection stimulates increased antibody affinity maturation that is unable to keep pace with viral replication and the cytokine storm. In this sense, CXCL13 could be used as a marker of SARS-CoV-2 disease severity. There is a precedent for the utility of CXCL13 as a biomarker that is predictive of immune activation during HIV exposure (8, 9, 20). This adds credibility and feasibility for this utility, but further studies are required to validate this approach. We have provided a schematic of how the CXCL13 response interplays with our other observations of SARS-CoV-2 immunity in Fig. 6.

To summarize, this study provides insight into the breadth of the immunological response against SARS-CoV-2. We demonstrated increasing antibody production to multiple SARS-CoV-2 antigens over the first 10 days of infection using a rapid-ELISA. Our results exhibit that patient mortality, sex, blood type, and age impact antibody production to SARS-CoV-2, adding to what is known about SARS-CoV-2 pathogenesis. Furthermore, lethal SARS-CoV-2 infection triggers a proinflammatory cytokine response, in combination with the secretion of several chemotactic agents. Interestingly, patients with lethal SARS-CoV-2 disease exhibited divergent cytokine production compared to patients with nonlethal disease. Finally, we discovered that a marker of germinal center activity (CXCL13) is upregulated in SARS-CoV-2 patients and that this upregulation is amplified in lethal disease. Ultimately, these studies help to elucidate the interplay between immunological responses to SARS-CoV-2 and identify a potential novel biomarker of COVID-19 severity.

## MATERIALS AND METHODS

### Patient sampling and analysis.

Clinical results of molecular FDA emergency use authorization (EUA)-approved assays (Abbott M2000, BD Max, and Cepheid GeneXpert) were reviewed by querying an electronic health record (HER; Epic, Verona, WI) at least 3 times a week, for admitted inpatients with positive or negative COVID-19/SARS-CoV-2 test results at West Virginia University Hospital (WVUH) (see all patient information in Data Set S1 in the supplemental material). All available residual serum and plasma clinical specimens (491 from 79 inpatients, collected from day 0 to day 55 post-symptom onset) were then retrieved, deidentified, and stored at −80°C. Electronic medical records were reviewed (IRB no. 2004976401) for symptoms, date of symptom onset, and patient demographic information (age, sex, and mortality). If no symptoms were recorded, the date of admittance was documented as date of symptom onset. Serological results from EUA-approved antibody testing (Abbott Architect) performed in the WVUH clinical laboratory and ABO blood type were documented when available. Patient specimens were deidentified by appropriately Collaborative Institutional Training Initiative (CITI)-trained clinical research staff before transfer to the WVU Vaccine Development center research laboratory for testing and aliquoting. Once specimens were thawed for testing, residual specimens were aliquoted into 1-ml cryovials in 0.5-ml increments and frozen at −80°C. No specimens underwent >3 free thaw cycles prior to testing to prevent degradation.

### Production of SARS-CoV-2 RBD protein.

Production of the SARS-CoV-2 RBD protein was done by transient transfection of the HEK293T cells with a pCAGGS mammalian expression vector containing an RBD construct with a C-terminal hexahistidine tag and codon optimized for mammalian expression (pCAGGS vector catalog number NR-52309; BEI Resources, Manassas, VA, USA). SARS-CoV-2 RBD protein was produced by transient transfection of HEK293T cells cultured in 300-cm^2^ flasks. Each flask with 60 to 80% confluent cells was transfected with 60 μg of plasmid DNA complexed with 120 μg of 25-kDa linear polyethylenimine (PEI) (Polysciences Inc., Warrington, PA, USA). The DNA/PEI complex was prepared by slowly adding the PEI solution (0.08 mg/ml in phosphate-buffered saline [PBS]) to the DNA solution (0.04 mg/ml in PBS) with continuous mixing followed by 10 min of incubation at room temperature; the DNA/PEI complex was then diluted with 45 ml of serum-free Dulbecco's modified Eagle medium (DMEM) and used to replace the fetal bovine serum (FBS)-supplemented DMEM in the flask. For the production of the SARS-CoV-2 N protein, HEK293T cells were harvested 72 h posttransfection and kept at −80°C until processing. For the production of the SARS-CoV-2 RBD protein, which is secreted into the culture medium, the cell culture medium was collected after 48 h, stored at 4°C, and replaced with fresh serum-free DMEM; after an additional 48 h, corresponding to 96 h posttransfection, the medium was collected, pooled with the 48-h-posttransfection medium, and stored at 4°C until processing. Five hundred milliliters of medium was supplemented with 500 U of Pierce universal nuclease (Thermo Fisher Scientific) and centrifuged at 4,000 × *g* for 20 min, and the supernatant was filtered through a 0.45-μm polyethersulfone (PES) membrane. The filtered medium was applied onto 5-ml HisTrap FF cartridges (GE Healthcare Bio-Sciences) installed on an AKTA purifier run with buffer A (20 mM NaH_2_PO_4_, 0.5 M NaCl, 10 mM imidazole, pH 7.4) and buffer B (20 mM NaH_2_PO_4_, 0.5 M NaCl, 500 mM imidazole, pH 7.4). The cartridge was then washed with 20 mM imidazole (98% A, 2% B), and the protein was eluted by linear gradient from 2% to 100% buffer B in 10 column volumes. Protein quality was checked by SDS-PAGE, and after dialysis in PBS, the concentration was estimated using the Coomassie protein assay and bovine gamma globulin as standard.

### SARS-CoV-2 ELISAs.

Upon receipt of patient samples, 100-μl aliquots were generated and heat inactivated at 56°C for 1 h while shaking at 500 rpm. Remaining samples were labeled and stored at −20°C or −80°C. When ready to assess antibody concentration, 20 μl of each sample was added to 100 μl of 1% nonfat dry milk diluted in PBS plus 0.1% Tween 20 (PBS-T) in the first row of 3 preblocked and coated enzyme-linked immunosorbent assay (ELISA) plates (Pierce part no. 15041): one coated with SARS-CoV-2 receptor RBD (2 μg/ml), one coated with N (Sino Biological part no. 40588-V08B) (1 μg/ml), and one coated with S1 (Sino Biological part no. 40591-V08H) (2 μg/ml). RBD used to validate the rapid-ELISA prior to serological analysis of patient samples was contributed by David Veesler for distribution through BEI Resources, NIAID, NIH [vector pcDNA3.1(−) containing the SARS-related coronavirus 2, Wuhan-Hu-1 spike glycoprotein receptor binding domain (RBD), NR-52422] (62). S1 was selected as an antigen to identify whether trends in antibody levels differed with the inclusion of a greater number of epitopes than RBD alone. Samples were diluted 5-fold down the plate. excluding the final row. which served as a negative control for each patient sample. A positive-control human monoclonal antibody against an individual antigen was run on each plate to ensure lot-to-lot consistency (human-anti-S1/RBD, Sino Biological part no. A02038 [HC2001]; rabbit anti-N, Sino Biological part no. 40143-R001). After sample loading, plates were incubated for 10 min at room temperature with shaking at 60 rpm. Plates were then washed four times with PBS-T. Secondary antibody buffer (100 μl of 1% milk diluted in PBS-T containing 1:500 goat anti-human-IgG-horseradish peroxidase [HRP]; Invitrogen part no. 31410) was added immediately following the washing procedure. The plates were incubated for 10 min at room temperature with shaking at 60 rpm. Plates were washed five times with PBS-T. SigmaFast *ortho*-phenylenediamine (OPD) substrate (Sigma part no. P9187) was prepared in MilliQ (18.2 MΩ × cm) water, and 100 μl was aliquoted into each well. Ten minutes after loading of the substrate, 25 μl of stop solution (3 N HCl) was added to end colorimetric development. The absorbance of the substrate in each well was measured on a Synergy H1 (BioTek) spectrophotometer at 492 nm. Antibody concentration was calculated based on area under the curve (AUC) analyses of *A*_492_ versus dilution factor plots for each sample.

### Cytokine quantification.

Serum cytokine concentrations of IL-1β, IL-2, IL-4, IL-5, IL-6, IL-8, IL-12, IL-13, IL-18, eotaxin, granulocyte-macrophage colony-stimulating factor (GM-CSF), GRO-α, IFN-γ, IP-10, MCP-1, MIP-1α, MIP-1β, RANTES, SDF-1α, and TNF-α were assessed using a human Th1/Th2 cytokine and chemokine 20-plex ProcartaPlex panel 1 (ThermoFisher part no. EPX200-12173-901) according to the manufacturer’s instructions. Serum samples (217 samples) were prepared for analysis by heating at 56°C for 1 h to inactivate SARS-CoV-2 virus. Samples were then centrifuged at 13,000 × *g* for 2 min to pellet aggregates. Samples (25 μl) were diluted 1:2 with universal assay buffer and incubated at room temperature on an orbital shaker at 500 rpm for 1 h. Select samples (based on sample quantity) were diluted 1:4 or 1:5 with the universal assay buffer, which was taken into account during analysis. A standard curve was generated using antigen standards provided by the manufacturer. Samples were resuspended in 120 μl wash buffer prior to running on a Magpix (Luminex) instrument, and 35 μl was analyzed per sample. Bead counts below 35 were insufficient for analysis and excluded from the analysis. The majority of cytokines appeared stable under these assay conditions although the final concentration may have been diminished due to the mandatory heat inactivation of the serum. This step was included for all samples and thus did not impact the comparison of one sample to another.

### CXCL13 quantification.

CXCL13 concentration was determined using a human BLC (CXCL13) ProcartaPlex Simplex kit (ThermoFisher part no. EPX01A-12147-901). Plates were coated with magnetic beads according to the manufacturer’s protocol. Plasma samples (25 μl, 217 samples) from patients were loaded onto coated plates and shaken for 1 h at 500 rpm at room temperature. Plates were washed 2 times with wash buffer while attached to the magnet before the addition of detection antibody. Samples were shaken (500 rpm) for 30 min at room temperature to allow for detection antibody binding. Plates were then washed 2 times with wash buffer while attached to the magnet. After washing, 50 μl of streptavidin-phycoerythrin (SAPE) was added to each well and the plates were shaken (500 rpm) for 30 min at room temperature. Finally, plates were washed 2 times with wash buffer attached to the magnet before the addition of 120 μl of reading buffer. Sample aliquots (35 μl) were read by the Luminex Magpix instrument with a 35-bead detection limit.

### Principal-component and heatmap analysis.

Serological data from patients tested for cytokine levels and antibody levels were pooled into Microsoft Excel and imported to ClustVis (63). Data were transformed by the ln(x) transformation provided in the webtool and grouped with a 95% confidence interval. Groups were based on patient SARS-CoV-2 status and outcome (survived versus deceased). Heatmap clustering was based on complete cytokine profile.

### Statistical analyses.

Statistical analyses were calculated in GraphPad Prism (version 8.3.0). Comparisons of two conditions were completed using two-tailed Student *t* tests or Welch *t* tests in cases where standard deviations were different between groups. Statistical significance of multiple variables was assessed using Brown-Forsythe and Welch’s one-way analysis of variance (ANOVA) followed by Tukey’s multiple-comparison test. Pearson correlation coefficients and *P* values were calculated in GraphPad Prism using the “Correlation” analysis. In all analyses statistical significance was determined to be *P* < 0.05.

10.1128/mSphere.01324-20.2 FIG S2 Anti-SARS-CoV-2 antibody response correlates directly with age. IgG production to RBD (A), N (B), or S1 (C) or surviving SARS-CoV-2-positive patients as correlated with their age in years. Correlation was assessed by calculating Pearson correlation coefficients. Download FIG S2, PDF file, 0.05 MB. Copyright © 2021 Horspool et al. 2021 Horspool et al. This content is distributed under the terms of the Creative Commons Attribution 4.0 International license.

10.1128/mSphere.01324-20.3 FIG S3 Cytokine response to SARS-CoV-2 infection. Concentrations of IP-10 (a and b), IL-18 (c and d), MIP-1β (e and f), MIP-1α (g and h), MCP-1 (i and j), IFN-γ (k and l), IL-6 (m and n), SDF-1 (o and p), IL-1β (q and r), IL-8 (s and t), eotaxin (u and v), IL-2 (w and x), IL-4 (y and z), IL-12 (aa and bb), RANTES (cc and dd), TNF-α (ee and ff), and GRO-α (gg and hh) in patient samples. Surviving, SARS-CoV-2^+^ patients who survived infection, Deceased, SARS-CoV-2^+^ patients who did not survive infection. Statistical significance was assessed between total average cytokine concentrations across days (reported in longitudinal graph legend) and between cytokine concentrations averaged between days 7 and 21 (reported on longitudinal figures and histograms). Significance between bars was determined with a two-tailed Welch *t* test. Significance against average cytokine concentration of healthy controls (dotted line) was assessed with a one-sample *t* test. *, *P* < 0.05; **, *P* < 0.01; ***, *P* < 0.001; ****, *P* < 0.0001. #’s represent significance between bars on histograms. Download FIG S3, PDF file, 0.4 MB. Copyright © 2021 Horspool et al. 2021 Horspool et al. This content is distributed under the terms of the Creative Commons Attribution 4.0 International license.

10.1128/mSphere.01324-20.4 FIG S4 CXCL13 production by SARS-CoV-2 patients. CXCL13 production by SARS-CoV-2 production is compared to anti-RBD, anti-N, or anti-S IgG quantity over the course of patient disease. Patient age, sex, and status (S, survived; D, deceased) are represented left of the patient data. Download FIG S4, PDF file, 0.1 MB. Copyright © 2021 Horspool et al. 2021 Horspool et al. This content is distributed under the terms of the Creative Commons Attribution 4.0 International license.

10.1128/mSphere.01324-20.5 FIG S5 N/RBD or N/S1 ratios are associated with disease severity. The antinucleocapsid AUC was divided by either the anti-RBD AUC (A) or anti-S1 AUC (B) of patient samples grouped by whether they survived (S) or did not survive (D) SARS-CoV-2 infection. Statistical significance was assessed with a two-tailed Welch *t* test. **, *P* < 0.01; ***, *P* < 0.001. Download FIG S5, PDF file, 0.04 MB. Copyright © 2021 Horspool et al. 2021 Horspool et al. This content is distributed under the terms of the Creative Commons Attribution 4.0 International license.

10.1128/mSphere.01324-20.6 FIG S6 Cytokine profile over time of patients infected with SARS-CoV-2. Cytokine production was plotted over time for all cytokines quantified in this study (left *y* axis, solid lines) in addition to anti-SARS-CoV-2 antibody production (right *y* axis, dashed lines) for a representative series of patients. Patient age, sex, and status (S, survived; D, deceased) are represented at top left of the patient data. Download FIG S6, PDF file, 0.4 MB. Copyright © 2021 Horspool et al. 2021 Horspool et al. This content is distributed under the terms of the Creative Commons Attribution 4.0 International license.
