# Supplementary figures and images for: Interplay of Antibody and Cytokine Production Reveals CXCL13 as a Potential Novel Biomarker of Lethal SARS-CoV-2 Infection
Source: mSphere. 2021 Jan 20;6(1):e01324-20. doi: 10.1128/mSphere.01324-20 (PMC7845617; doi:10.1128/mSphere.01324-20)

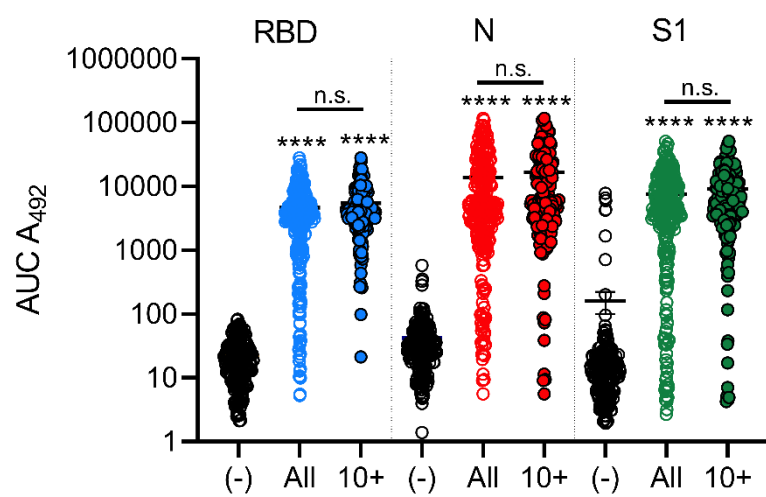

Supplementary Figure 1

Supplement: FIG S1 [file mSphere.01324-20-sf001.pdf]

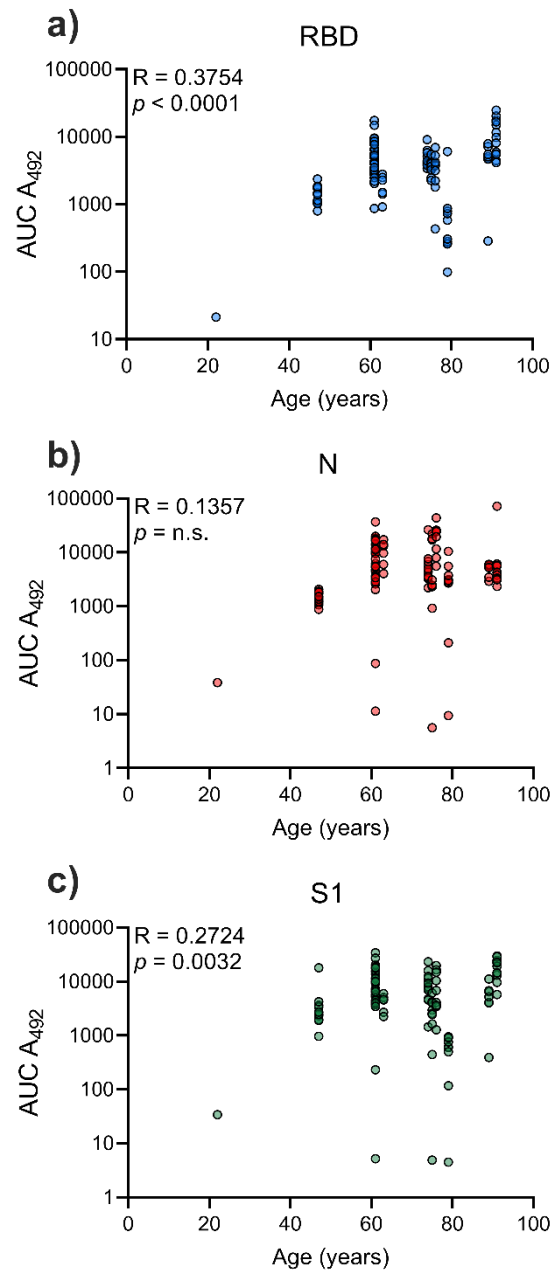

**Supplementary Figure 2**

Supplement: FIG S2 [file mSphere.01324-20-sf002.pdf]

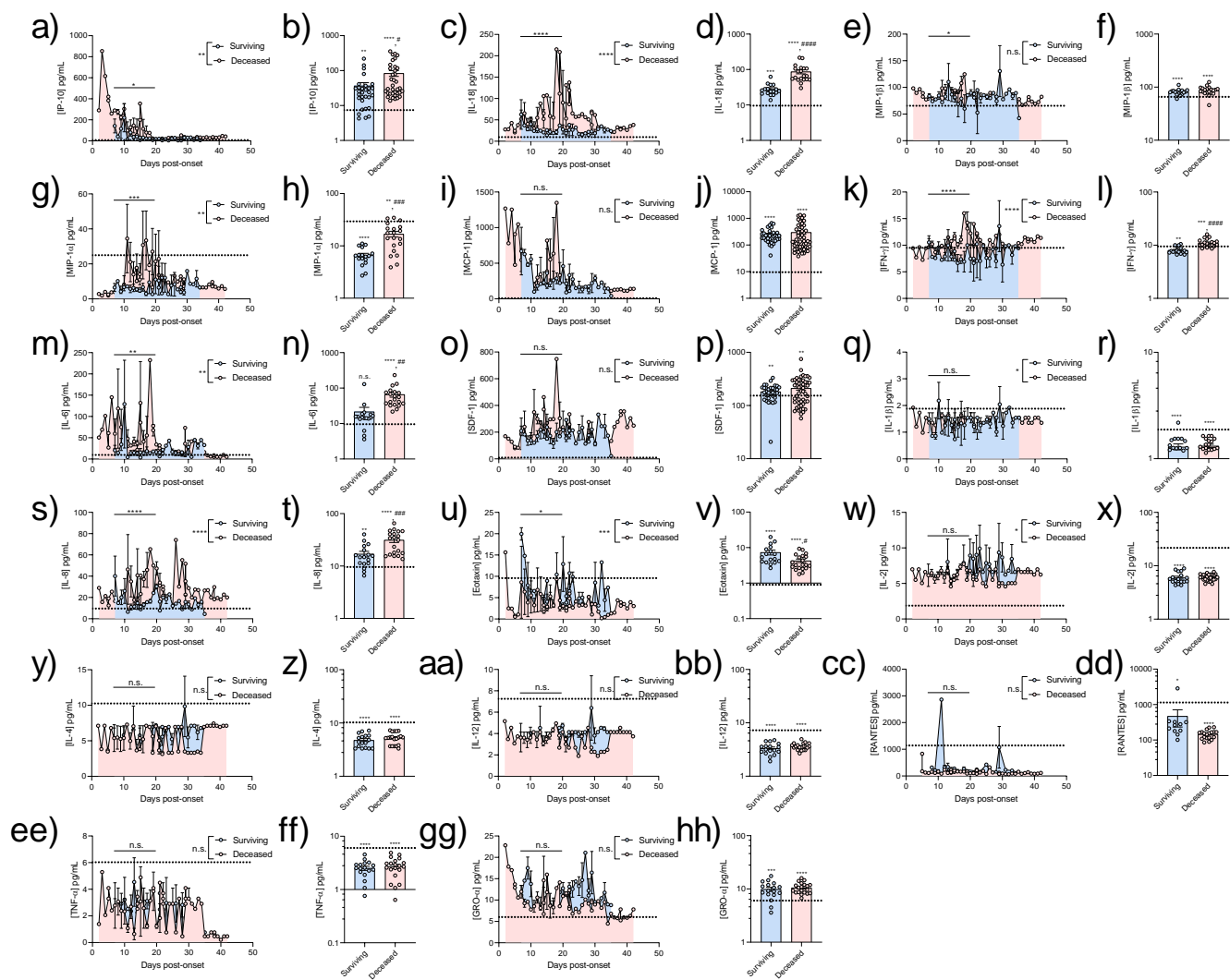

Supplement: FIG S3 [file mSphere.01324-20-sf003.pdf]

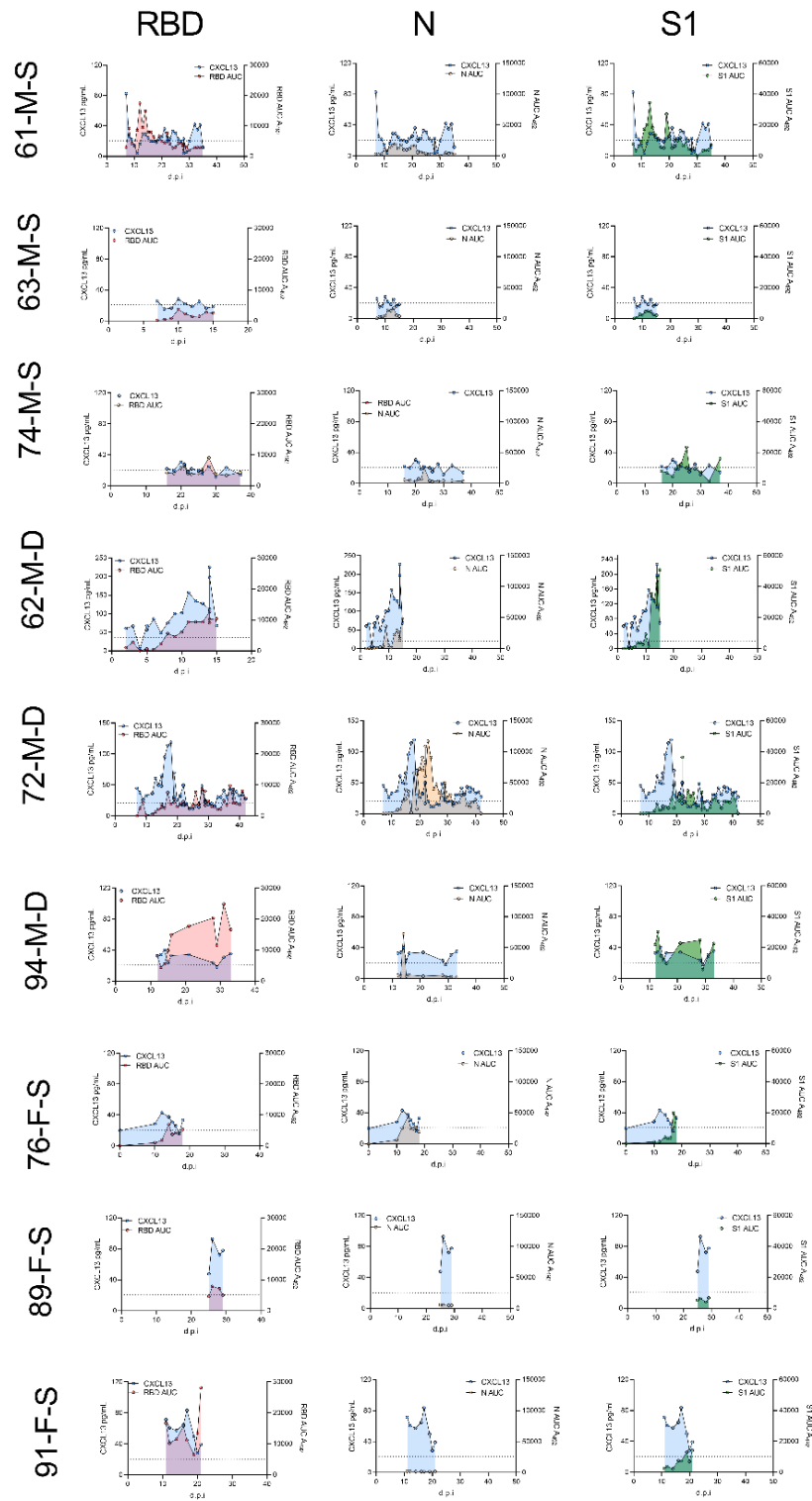

Supplementary Figure 4

Supplement: FIG S4 [file mSphere.01324-20-sf004.pdf]

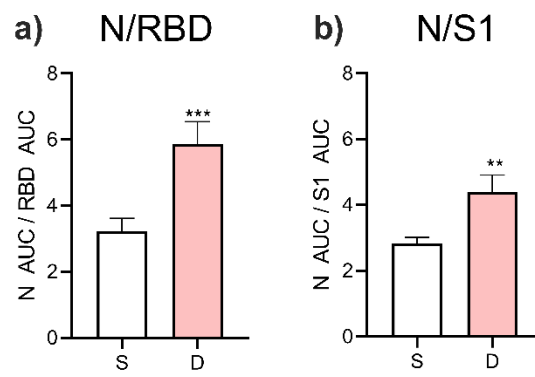

**Supplementary Figure 5**

Supplement: FIG S5 [file mSphere.01324-20-sf005.pdf]

## Male Survivors

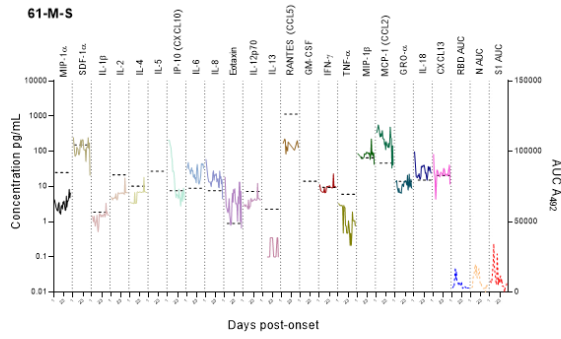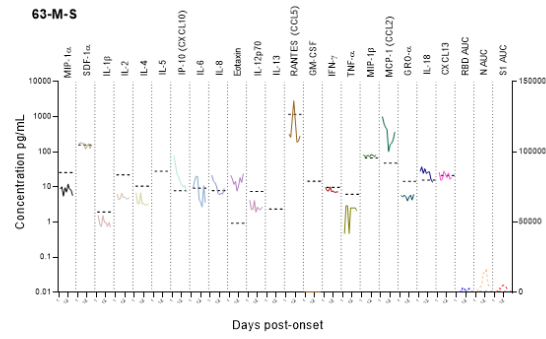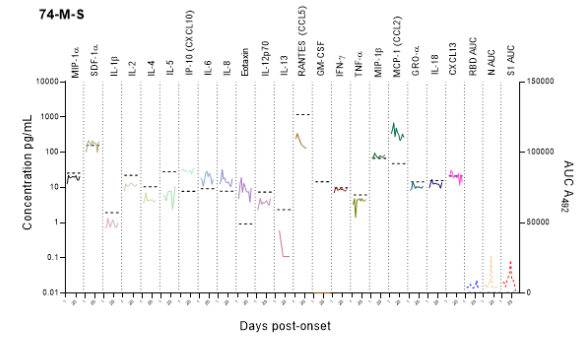

## Deceased

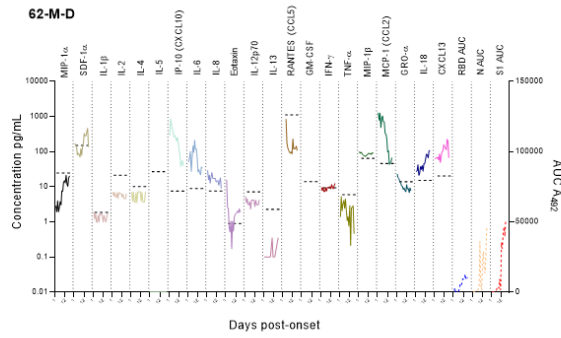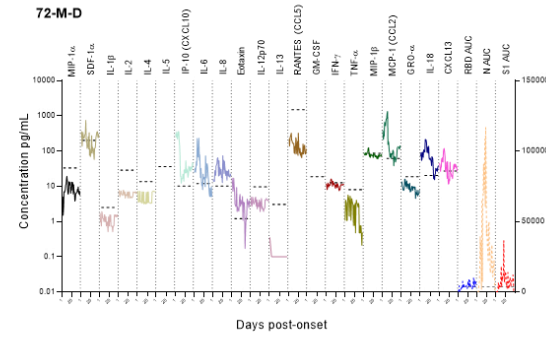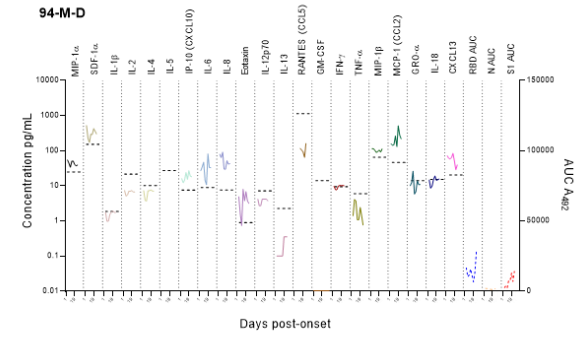

## Female Survivors

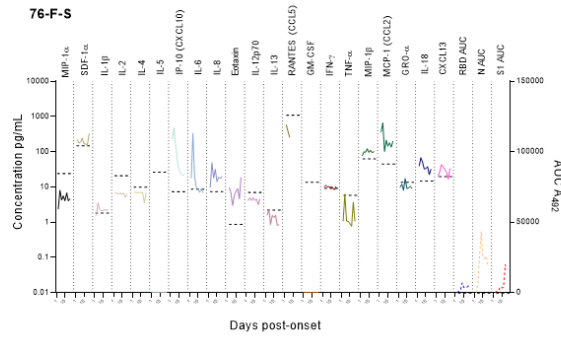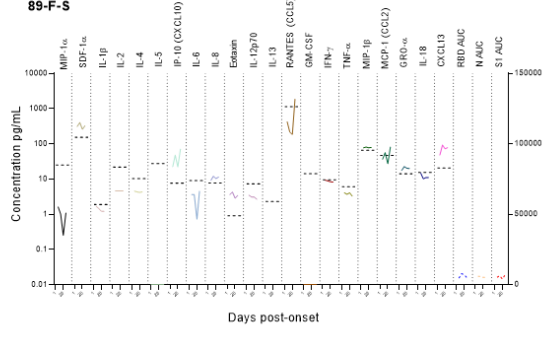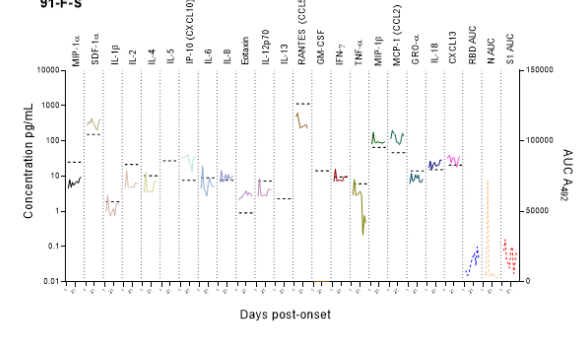

Supplementary Figure 6

Supplement: FIG S6 [file mSphere.01324-20-sf006.pdf]
