# Supplementary material for: Interplay of Antibody and Cytokine Production Reveals CXCL13 as a Potential Novel Biomarker of Lethal SARS-CoV-2 Infection
Source: mSphere. 2021 Jan 20;6(1):e01324-20. doi: 10.1128/mSphere.01324-20 (PMC7845617; doi:10.1128/mSphere.01324-20)
Supplement: TABLE S1 [file mSphere.01324-20-st001.pdf]

Supplementary Table 1

Demographics	Age (yrs)	Outcome D/S	Sex (F/M)	SARS-CoV-2 (+/-)
Average	61.20	12.66	40.51	18.99
SD	20.20	0.33	N/A	N/A
Patient n	79	79	79	79
