# Supplementary material for: Interplay of Antibody and Cytokine Production Reveals CXCL13 as a Potential Novel Biomarker of Lethal SARS-CoV-2 Infection
Source: mSphere. 2021 Jan 20;6(1):e01324-20. doi: 10.1128/mSphere.01324-20 (PMC7845617; doi:10.1128/mSphere.01324-20)
Supplement: TABLE S2 [file mSphere.01324-20-st002.pdf]

**Supplementary Table 2 | Accuracy of SARS-CoV-2 rapid-ELISA assay.**

| True (+) | False (+) | True (-) | False (-) |
|---|---|---|---|
| 196 | 0 | 51 | 1 |
| Total Samples | 247 | | |
| PPV | 99.5% | | |
| NPV | 100% | | |
